# Racial Categorization Predicts Implicit Racial Bias in Preschool Children

**DOI:** 10.1111/cdev.12851

**Published:** 2017-06-12

**Authors:** Peipei Setoh, Kristy J. J. Lee, Lijun Zhang, Miao K. Qian, Paul C. Quinn, Gail D. Heyman, Kang Lee

**Affiliations:** ^1^ Nanyang Technological University; ^2^ University of Toronto; ^3^ Hangzhou Normal University; ^4^ University of Delaware; ^5^ University of California, San Diego; ^6^ Zhejiang Normal University

## Abstract

This research investigated the relation between racial categorization and implicit racial bias in majority and minority children. Chinese and Indian 3‐ to 7‐year‐olds from Singapore (*N *=* *158) categorized Chinese and Indian faces by race and had their implicit and explicit racial biases measured. Majority Chinese children, but not minority Indian children, showed implicit bias favoring own race. Regardless of ethnicity, children's racial categorization performance correlated positively with implicit racial bias. Also, Chinese children, but not Indian children, displayed explicit bias favoring own race. Furthermore, children's explicit bias was unrelated to racial categorization performance and implicit bias. The findings support a perceptual–social linkage in the emergence of implicit racial bias and have implications for designing programs to promote interracial harmony.

Implicit racial bias has broad personal and societal consequences in all spheres of human life including education, politics, healthcare, employment, justice, finance, and even dating (e.g., Chugh, 2004). Because this negative bias is firmly entrenched and difficult to change by adulthood, it is crucial to understand its emergence in early childhood so as to develop prevention and intervention strategies that can be used early in life.

A framework for thinking about the ontogenetic emergence of implicit racial bias is the perceptual–social linkage hypothesis (Lee, Quinn, & Heyman, 2017). This hypothesis proposes that implicit bias in early childhood results from two early tendencies: the tendency to categorize and the tendency to form relatively more positive associations with familiar categories. In the present research, we test one central prediction derived from this hypothesis: that young children's tendency to categorize faces by race is predictive of their level of implicit racial bias.

The perceptual–social linkage hypothesis is based on a number of findings regarding the development of cross‐race face processing and implicit bias from the last decade. First, most children, especially those belonging to racial majorities, have asymmetrical visual exposure to faces of different races from birth: they see own‐race faces far more frequently than other‐race faces (Rennels & Davis, 2008; Sugden, Mohamed‐Ali, & Moulson, 2014). Due to this perceptual asymmetry, infants from 9 months onward not only readily classify own‐race faces into one category and other‐race faces into another category (Anzures, Quinn, Pascalis, Slater, & Lee, 2010; Quinn, Lee, Pascalis, & Tanaka, 2016) but also process own‐race faces at the individual level more than other‐race faces (Kelly et al., 2007, 2009). The own‐race face recognition advantage emerges early in infancy and is present throughout childhood (Anzures et al., 2010; Lee, Quinn, Pascalis, & Slater, 2013). Second, training infants to individuate other‐race faces leads to significant improvement in other‐race face recognition and thus reduces cross‐race face processing differences (Anzures et al., 2012; Heron‐Delaney et al., 2011). Third, training children to individuate other‐race faces leads to a significant reduction in implicit racial bias (Xiao et al., 2015). Based on these findings, the perceptual–social linkage hypothesis posits that early asymmetry in own‐ versus other‐race face experience will have not only perceptual consequences in terms of face recognition and categorization but also social consequences in terms of racial biases. More importantly, this hypothesis postulates that the two kinds of consequences are related—perceptual processing differences trickle downstream to influence the meaning attached to social stimuli (Quinn et al., 2013).

Early theories of implicit cognition suggest that implicit bias arises from automatic nonconscious associations that differ in valence across well‐established categories stored as mental structures in the mind (Devine, 1989). In this article, we define implicit racial bias as the low‐level evaluative associations that an individual holds toward different race groups. To the extent that social categories and their accompanying positive or negative associations are spontaneously activated by perceptual input, the perceptual readiness to classify people into racial categories presents a minimal basis for the development of implicit racial bias. In addition, because children are frequently exposed to same‐race individuals in their immediate environment, and have ample opportunities to engage in positive reciprocal interactions with same‐race members of their family (e.g., Kim & Johnson, 2014; Malatesta & Haviland, 1982), they tend to develop positive associations for their own‐race category. In contrast, the lack of positive and repeated exposure to other‐race individuals, as well as children's general wariness of unfamiliar social stimuli (e.g., Bronson, 1972), results in a poverty of positive associations with other‐race categories.

Developmental theories and previous research have suggested that social categorization is a sufficient and necessary antecedent to intergroup bias and discrimination (Allen & Wilder, 1975; Bigler & Liben, 2006; Billig & Tajfel, 1973; Rennels & Langlois, 2014; Tajfel & Turner, 1979). Regarding explicit racial bias, a few studies have found it to be positively associated with children's social categorization skills (e.g., Bigler & Liben, 1993). In contrast, regarding implicit racial bias, only one study has administered continuous measures of individual differences in categorization performance and examined their correlates with the strength of implicit social bias (Dunham, Chen, & Banaji, 2013). In this earlier study, children's tendency to categorize racially ambiguous synthetic happy versus angry faces was used as a measure of their implicit racial bias. Racial majority children between 4 and 12 years of age were observed to categorize racially ambiguous happy faces as own race and racially ambiguous angry faces as other race. As a control, racially unambiguous faces were included and used to measure children's tendency to categorize such faces by race. It was found that racial majority children's categorization performance predicted their implicit racial bias: Those who could accurately categorize racially unambiguous faces by race had greater implicit bias than those who could not. This finding seems to provide direct support for the perceptual–social linkage hypothesis regarding the association between perceptual categorization and implicit racial bias. However, one can argue that this finding reflects children's performance in a single task (i.e., classification of faces as own or other race) that measured both children's categorization (with neutral faces) and implicit bias (with happy or angry faces). Furthermore, this finding might also have been influenced by use of synthetic faces that could have appeared artificial and unnatural to younger children.

To avoid these potential issues, in this study, we used separate and distinctively different tasks to measure racial categorization and implicit racial bias. In addition, we used color photographs of natural faces as stimuli. More specifically, we measured the tendency of preschool children to categorize faces by race using photographs of real own‐ and other‐race faces. We also measured their implicit racial bias, using a cross‐validated preschooler‐friendly implicit racial bias task (Qian et al., 2016) that is based on the traditional implicit association test (IAT). We chose to test preschool‐age children because we expected them to exhibit higher variability in racial categorization performance compared to older children and adults, who we expected to be more likely to perform at ceiling levels (Anzures et al., 2013).

We recruited preschool children from Singapore. One reason that we selected Singapore is because it is a heterogeneous, multiethnic society: The population consists of about 74.3% Chinese, 13.3% Malay, 9.1% Indian, and 3.2% other ethnicities (Singapore Department of Statistics, 2016). We were interested in testing the perceptual–social linkage hypothesis in such a diverse population, under the assumption that diverse exposure to individuals from other races would lead to greater variability in categorizing faces by race as well as greater individual differences in implicit racial bias. Such variabilities should allow for more effective assessment of the relation between these two constructs. This consideration follows from prior research suggesting little variability in racially homogenous locations, where preschool‐age children tend to show robust implicit racial bias against other‐race individuals (Dunham, Baron, & Banaji, 2006; Qian et al., 2016) and near ceiling levels in race classification accuracy (see Anzures et al., 2013 for a review). We also expected to see variability in racial categorization performance and implicit bias due to the high level of integration between diverse groups in Singapore. Such integration is promoted by official government policies and other institutions. For example, residential neighborhoods of government housing, where about 80% of the population lives, are required to fulfill a racial mix that is representative of national racial proportions with the aim of fostering interracial interaction and cohesion.

Although almost all children in Singapore are likely to have extensive exposure to individuals from other races, the nature of this exposure differs in important ways that are likely to affect how children process race‐related information. For example, there is variability in home care arrangements (e.g., whether the child's family has a live‐in other‐race domestic helper), in networks of familial friends (e.g., whether parental friends are predominately own race or multiracial), and in out‐of‐home care arrangements (e.g., whether the child is attending a public or a private school, with most public schools being more racially diverse than private ones). These additional considerations led us to expect even more strongly that Singaporean children would have sufficient variability in both racial categorization performance and implicit racial bias, thereby allowing for examination of their relation, the focus of the present study.

We investigated the hypothesis that racial categorization performance would be related to implicit bias by testing Chinese and Indian preschool children in Singapore with Chinese and Indian faces. Choosing these two racial groups has several advantages. First, there are clear perceptual differences between Chinese and Indians in terms of facial physiognomy such as skin tone, face shape, and size and shape of key facial features (e.g., nose). Second, the Chinese and Indian populations are highly comparable in Singapore society. For example, they have similar socioeconomic status, with gross monthly income within 1.5% of each other in all income brackets except at the highest income bracket of USD $8,640 and above monthly, where there are more Indians than Chinese (12.97% vs. 9.87%, respectively; Singapore Department of Statistics, 2016). This is in contrast to the Malays, another large minority group in Singapore, who have greater representation in income brackets of USD $3,400 a month and below compared to Chinese and Indians, and only 1.41% are in the highest income bracket. For educational attainment, 82% of Chinese and 63% of Indians of 20 years of age and older have attained a university education, compared to only 46% of Malays. Chinese and Indians also hold similar levels of political power; the Singapore cabinet is made up of 70% Chinese, 20% Indian, and 10% Malay cabinet ministers. The 20% level of representation for Indian cabinet ministers is noteworthy given that the population comprised only 9.1% Indians. Although Singapore has legislated structural and institutional equality for different ethnicities, and there exists outward racial harmony and social cohesion, some argue that minorities may still feel discriminated against in their everyday experiences (Velayutham, 2009). In a recent survey, only 17% of Singapore Chinese respondents ever felt racially discriminated, compared to 33% of Malay and 36% of Indian respondents (Mathews, 2016). This possible disconnect between egalitarian ideals and reality makes it all the more pertinent to study implicit and explicit racism within this population.

The similarity in social status between target racial groups in this study is important in light of evidence that social status moderates displays of racial bias. Existing studies have demonstrated that children associate different racial groups with wealth differences (Olson, Shutts, Kinzler, & Weisman, 2012) and exhibit implicit racial bias favoring their own race only when the racial out‐group is perceived as lower in social status but not when the racial out‐group is of higher social status (Bigler, Brown, & Markell, 2001; Dunham, Baron, & Banaji, 2007; Shutts, Kinzler, Katz, Tredoux, & Spelke, 2011). This sensitivity to the social status of racial out‐groups also increases with age, such that older children become less likely to display implicit bias in favor of a high‐status racial out‐group, whereas implicit bias against a low‐status racial out‐group remains stable over time (Dunham et al., 2006). Children from lower status racial groups, who express explicit preference for high status (operationalized in terms of wealth), display implicit bias favoring the high‐status racial out‐group (Newheiser & Olson, 2012). The selection of majority (Chinese) and minority (Indian) race groups that are similar in socioeconomic status in Singapore therefore allows us a unique opportunity to compare racial biases between a majority and minority ethnic group with similar social status within the same society.

The present study, in two experiments, assessed children's categorization of faces by race using a racial categorization task and their implicit racial bias using the implicit racial bias test (IRBT; Qian et al., 2016). Children were presented with a random sequence of Chinese and Indian faces in the racial categorization task and were asked to identify whether each face was a Chinese or Indian face. This task measures how accurately children could categorize Chinese and Indian faces into their respective racial groups. During the IRBT, children were presented with photos of faces and were asked to match own‐ and other‐race faces with either smile or frown graphic buttons. This procedure provided a child‐friendly adaptation of the IAT that measures how fast participants associate attributes of positive and negative valence to own versus other race (Greenwald, McGhee, & Schwartz, 1998; Greenwald, Nosek, & Banaji, 2003). Notably, the IRBT was designed such that pictorial stimuli are used instead of words, and hence performance is less contingent on reading and word comprehension abilities compared to the traditional IAT used with adults, as young children may find such language demands challenging (Danziger & Ward, 2010).

The IRBT is presented to children in the form of a rule‐based matching game, in which participants have to match either a cartoon smile or a cartoon frown with own‐race or other‐race faces by pressing the cartoon faces embedded as response buttons on the screen. The IRBT is similar to the preschool IAT (PSIAT) that has been used in previous studies examining other forms of implicit bias with preschoolers (Cvencek, Greenwald, & Meltzoff, 2011; Thomas, Burton Smith, & Ball, 2007), except that unlike in the PSIAT, visual reminders of the prevalent matching rule (e.g., smile and own‐race exemplars on one side, frown and other‐race exemplars on the other side for congruent trials) are not presented on either side of the screen where the response buttons are embedded. An advantage of not including visual reminders of the matching rule in the IRBT is that children are required to make independent judgments of the race of each target face without reference to the exemplars, which is relevant to the current study given that the acquisition of racial categories takes on a more varied developmental course than the acquisition of gender or weight categories that were of interest in the prior PSIAT studies. Furthermore, a pioneering study using the IRBT (Qian et al., 2016) obtained low error rates (< 10%) and a moderate effect size (Cohen's *d *=* *.40) on the measure, providing support for its effectiveness and appropriateness in assessing implicit racial biases among preschoolers.

Although our primary interest was in children's performance in categorizing faces by race and its association with implicit racial bias, we also assessed children's explicit racial bias to determine the specificity of any potential relation between categorization performance and implicit racial bias, and whether children's racial categorization performance is also associated with their explicit racial bias. Two possibilities exist. According to the existing developmental theories concerning social biases (e.g., Bigler & Liben, 2006), children's race categorization performance should be associated positively with explicit racial bias. This is based on the findings of several existing studies examining the relation between children's social classification skills and their explicit social biases. For example, Rennels and Langlois (2014) found that the number of dimensions (e.g., gender, race, and attractiveness) on which children could classify faces predicted their explicit gender bias. Moreover, Bigler and Liben (1993) demonstrated that school‐aged children's ability to categorize people on various dimensions, such as race, gender, and age, predicted their explicit racial stereotypes.

An alternative possibility may exist regarding the perceptual categorization of faces by race and explicit racial bias. Existing evidence suggests that socialization practices, rather than perceptual processes, play a formative role in the development of explicit racial bias, such that children pick up social norms related to overt attitudes, expressions, and behaviors toward other‐race individuals from their parents, and reproduce these norms in a conscious and deliberate manner (Degner & Dalege, 2013; Miklikowska, 2016). Therefore, on the assumption that implicit and explicit racial biases operate on different sets of ontogenetic pathways, children's perceptual categorization of faces by race might only be predictive of implicit bias.

Overall, then, in accord with the perceptual–social linkage hypothesis, we predicted that Chinese and Indian preschoolers in Singapore would have varying abilities to categorize faces by race as well as varying degrees of implicit racial bias. Finally, we predicted that children's performance in categorizing faces by race would increase significantly with increased age, and this performance would be significantly correlated with their implicit racial bias, but not with explicit racial bias.

## Experiment 1

### Method

#### Participants

The sample consisted of 87 preschoolers aged 36–76 months (50 male, 37 female; *M*_age_ = 57.56 months, *SD *= 10.58 months). Data collection took place over the period from December 2015 to February 2016. All participants were ethnic Chinese, and 65.5% had parents who were both Singaporeans, whereas the remaining 34.5% had at least one parent who was a noncitizen but a permanent resident of Singapore. Parental consent was obtained prior to the testing sessions. Participants were recruited from preschools located in the northern and eastern regions of Singapore, within housing estates mainly occupied by middle‐class families. In accordance with the Ethnic Integration Policy in Singapore, public housing apartments must abide by an ethnic quota that specifies the permissible proportion of apartments in each neighborhood that can be occupied by members of different race groups. The ethnic quota is matched closely to the overall ethnic composition in Singapore. As priority for enrollment is given to families who live nearby, homes of the preschoolers tend to be situated within the vicinity of their preschool. Consent forms were distributed to the preschools, and each child whose parents gave consent was tested individually in a short session, which took place in a quiet room at the child's preschool.

#### Materials and Procedure

Each child was seated on a chair within comfortable reach of a Microsoft Surface Pro 3 tablet (Microsoft Corporation, Redmond, WA, USA) that was used to present face stimuli and collect reaction time data. A female Chinese experimenter sat at the right of the child and provided clear instructions for each task. Testing sessions were conducted in each participant's dominant language, either English or Mandarin Chinese.

##### Racial categorization task

Participants were presented with four different Chinese faces (two female) and four different Indian faces (two female) in a random sequence. The faces were selected from an existing face database (Yap, Chan, & Christopoulos, 2016). All photos were standardized at 480 pixels (17 cm) in width and 600 pixels (21 cm) in height, with a resolution of 72 pixels per in. The faces were frontal views without glasses, facial hair, or makeup, and were overlaid with the same elliptical shape such that hair was concealed. All faces depicted neutral expressions. On each trial, participants were asked to identify which racial group each face belonged to (*Is this a Chinese face or an Indian face?*) and were given feedback on whether their responses were correct, with a red star indicating a correct response and a black X indicating an incorrect response. Feedback ensured that children understood that there were right and wrong answers and therefore discouraged random responding. As there were only eight trials, we did not expect a learning effect. Racial categorization scores were then computed based on the total number of faces each participant was able to correctly categorize into their respective racial groups.

##### Implicit racial bias measure

We adapted the Chinese‐Black IRBT (Qian et al., 2016) to assess children's implicit racial bias toward Chinese and Indians. The IRBT in our study was identical to the Chinese‐Black IRBT used by Qian et al. (2016), except that our face stimuli consisted of Chinese and Indian faces. A set of 24 photographs of Chinese faces (12 female) and 24 photographs of Indian faces (12 female) were selected from the same face database mentioned earlier (Yap et al., 2016). All faces depicted neutral expressions. Photos were unique across trials and were standardized to the same dimensions specified for the racial categorization task.

We chose adult faces from a sample of high‐quality Asian photos (Yap et al., 2016). The mean age of all face stimuli used was 22.69 years old, with a standard deviation of 2.68 years, and there was no significant age difference between the Chinese faces (*M *=* *22.42) and Indian faces (*M *=* *22.96), *t*(46) = −0.69, *p *=* *.49. Both adult (Qian et al., 2016; Xiao et al., 2015) and child (Baron & Banaji, 2006; Dunham et al., 2006, 2007) face stimuli have been used in previous investigations of implicit racial bias among children, and congruent findings from those studies suggest that children's performance on the IAT did not differ based on the age group of face stimuli used in testing. A pool of 37 undergraduates rated the face stimuli we used on a 7‐point Likert scale of attractiveness (1 = *very unattractive*, 7 = *very attractive*). A one‐way repeated measures analysis of variance (ANOVA) determined that the Chinese and Indian photos did not differ significantly in perceived attractiveness, *F*(1, 36) = 0.13, *p *=* *.73, partial η^2^ = .003.

On each trial, a face was presented in the middle of the screen, with a cartoon smile and a cartoon frown positioned near the bottom at the two sides of the screen. Data on participant response latencies were collected with E‐prime 2.0 Professional (Psychology Software Tools, 2012, Sharpsburg, PA). There were eight practice trials and 20 test trials each for congruent and incongruent blocks. In the congruent blocks, participants were presented with photographs of Chinese faces and were instructed to touch the cartoon smile when they saw such faces. They were also presented with photographs of Indian faces, and were instructed to touch the cartoon frown when they saw these faces. In the incongruent blocks, participants were presented with photographs of Chinese faces, and were told to touch the cartoon frown when they saw such faces; additionally, they were presented with photographs of Indian faces and were told to touch the cartoon smile when they saw such faces. The exact instructions for the congruent blocks were as follows:

For this game, you will see a Chinese face or an Indian face in the middle of the screen. There will also be a happy face and a sad face at the two corners of the screen. Rule number one is that when you see a Chinese face, you must press the happy face; and when you see an Indian face, you must press the sad face. Rule number two is that you must hold the tablet like this (*experimenter demonstrates how to hold the tablet*) and use only your thumbs to press the happy or sad faces. Rule number three is that you must play this game as fast as you can.

The sequence of congruent and incongruent blocks, as well as the left–right display position of the cartoon smile and frown, was counterbalanced across participants. The cartoon smile and frown faces were always shown on the same sides within each set of trials. There were two blocks of trials (congruent, incongruent) with a practice and test phase within each block, that is, four distinct sets of trials in total. The rationale for not randomizing the placement of response buttons within each set of trials is to avoid confusing the children as such random variation would demand additional cognitive resources to constantly update and switch between positional representations of the response buttons from trial to trial, which could be a confounding influence on accuracy and response latency. For each trial, participants were given feedback on whether they had responded correctly, with a red star indicating a correct response and a black X indicating an incorrect response.

To ensure that the rules were correctly remembered, participants were quizzed on them before starting on practice and test trials. Practice trials served as a warm‐up for participants to practice applying the rules. During the practice phase, the experimenter provided guidance if necessary. However, during the test phase, participants had to complete all trials independently, with no feedback, instructions, or any form of help from the experimenter. Between congruent and incongruent blocks, participants were given a short break of 5 min to rest.

##### Explicit racial bias measure

The measure of explicit racial bias was a choice task adapted from Qian et al. (2016). In the choice task, the experimenter read five brief scenarios to the children. The children were asked to choose between a Chinese and an Indian adult to be their swimming coach in Scenario 1, their dance teacher in Scenario 2, their drawing teacher in Scenario 3, their music teacher in Scenario 4, and their doctor in Scenario 5. An example is, “Next year, your mother will take you to a dance class. You can choose one person as your dance teacher. Which one would you like to choose?” For each scenario, children were simultaneously presented with photos of a Chinese adult and an Indian adult matched to their gender. The left–right display position of Chinese and Indian faces was counterbalanced across participants on Scenario 1 and then reversed for each subsequent scenario. Face stimuli consisted of 10 Chinese photos (5 female) chosen from the face database (Yap et al., 2016), and 10 Indian photos (5 female) selected from Internet search engines. All faces depicted neutral expressions, and extra care was taken to ensure that Chinese and Indian faces were comparable. The five different female (or male) faces from each race were presented across the five scenarios in a randomly determined order. All faces were novel and not used in previous tasks. A pool of 37 undergraduates rated both sets of faces for attractiveness. A one‐way repeated measures ANOVA indicated that the Chinese and Indian photos did not differ significantly in perceived attractiveness for either male faces, *F*(1, 36) = 0.39, *p *=* *.54, partial η^2^ = .01, or female faces, *F*(1, 36) = 2.36, *p *=* *.13, partial η^2^ = .06.

It should be noted that different faces were used in each of the above‐mentioned tasks so that they were all novel to the children, thus preventing stimulus familiarity from affecting the results.

### Results

#### Racial Categorization Task

Children's average score on the racial categorization task (*M *=* *5.93, *SD* = 1.88) was significantly higher than expected by chance (test value = 4), *t*(86) = 9.59, *p *<* *.001, Cohen's *d *=* *1.03. This result suggests that Chinese preschoolers were able to correctly categorize faces based on race significantly above chance level.

To determine if there was a learning effect due to feedback on the task, a generalized linear mixed model analysis (GLMM) was conducted on children's categorization performance across the eight trials using the statistical package *lme4* (Bolker et al., 2009) in R statistical software (version 0.99.902; RStudio Team, 2015). Children's accuracy was coded as a binary response (0 = incorrect, 1 = correct), and the model included trials as the fixed effect, with child ID entered as a random effect to account for repeated measures. There was no evidence of improved performance across trials, *b* = −.06, *p *=* *.18.

#### Implicit Racial Bias

To measure implicit racial bias, we computed *D* scores for each participant by obtaining the difference between averaged response latencies of congruent and incongruent test blocks divided by the standard deviation of response latencies across the two blocks (Greenwald et al., 2003). Following the recommendations of Greenwald et al. (2003), each error trial was replaced with the mean response latency of correct responses in the corresponding block plus a 600 ms penalty. In accord with the methods of previous child IAT studies (e.g., Cvencek et al., 2011), participants with ≥ 10% of responses faster than 300 ms, an error rate > 60%, or an average response latency 3 *SD* above the mean response latency of the whole sample were excluded. In accord with these criteria, data from three participants were omitted, leaving 84 participants for subsequent analyses.

A one‐sample *t* test revealed that the mean *D* score differed significantly from the no‐bias score of zero, *D *=* *.19 (*SD* = 0.62), *t*(83) = 2.85, *p *=* *.006, Cohen's *d *=* *.31. The positive mean *D* score indicates that children were faster at associating Chinese faces with smile stimuli and Indian faces with frown stimuli on the congruent trials than they were at associating Chinese faces with frown stimuli and Indian faces with smile stimuli on the incongruent trials. This finding suggests that Chinese Singaporean preschoolers as a group have a pro‐Chinese implicit racial bias.

Additionally, *D* scores were computed separately for male and female face stimuli. A one‐way repeated measures ANOVA found no significant difference between *D* scores for male and female faces, *F*(1, 81) = 0.03, *p *=* *.87, partial η^2^ = .001.

#### Explicit Racial Bias

Choice scores were calculated as the proportion of trials on which each participant chose Chinese over Indian as their potential interaction partners. A one‐sample *t* test using the test value of 0.5 indicated that the children favored Chinese over Indian on a significantly greater proportion of trials (*M *=* *0.66, *SD* = 0.28) than chance, *t*(86) = 5.45, *p *<* *.001, Cohen's *d *=* *.58. This finding suggests that Chinese Singaporean preschoolers as a group have a pro‐Chinese explicit racial bias.

We further examined choice patterns on each scenario, by conducting binomial tests on choice responses in each scenario. Children were significantly more likely to favor Chinese (own race) over Indian (other race) in all scenarios, binomial *p*s < .05, with the exception of Scenario 4 (choice of a music teacher) where they exhibited no significant explicit preference for own race over the other race, binomial *p *=* *.09.

#### Hierarchical Multiple Regression for Racial Categorization

Hierarchical multiple regression was conducted with racial categorization scores entered as the dependent variable. The data met assumptions of independent and normally distributed errors, homogeneity of variance, and linearity, and contained no outliers or multicollinearity between predictors. In the first step of the regression model, gender was entered as a predictor. It accounted for a nonsignificant proportion of variance, Δ*R* ^2^ = .01, *F*(1, 85) = 1.22, *p *=* *.27. Entering age in the second step explained an additional 24% of the variance in racial categorization scores, and this increase in explained variance was significant, *F*(1, 84) = 26.96, *p *<* *.001. Age contributed significantly to the prediction of racial categorization scores, β = .49, *p *<* *.001, part correlation = .49.

#### Hierarchical Multiple Regression for Implicit Racial Bias

To identify the predictors of implicit racial bias, hierarchical multiple regression was conducted with *D* scores entered as the dependent variable. Checks were made to ensure that the data met all assumptions for the analysis. An examination of standard residuals revealed that the data contained no outliers (std. residual min = −2.24, std. residual max = 2.89). Collinearity statistics of all predictor variables across all models were within acceptable limits (tolerance values > .1; Variance Inflation Factors (VIFs) < 10). The data also met the assumption of independent errors (Durbin–Watson value = 1.83). In addition, scatter plots of standardized residuals indicated that assumptions of normally distributed errors, homogeneity of variance, and linearity were satisfied.

**Figure 1:**
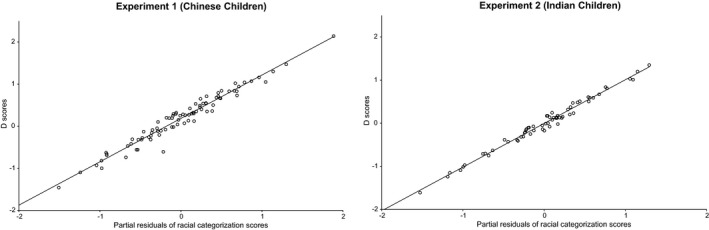
Partial regression plots of Experiment 1 (a, on left) and Experiment 2 (b, on right) depicting the prediction of *D* scores from racial categorization scores, after accounting for all other predictor variables.

Table 1 provides a summary of results from the hierarchical multiple regression analysis. Gender was entered as a predictor in the first model. It accounted for a nonsignificant proportion of variance, Δ*R* ^2^ = .001, *F*(1, 82) = 0.11, *p *=* *.74. Entering age in the second step explained an additional 7.9% of the variance in *D* scores, and this increase in explained variance was significant, *F*(1, 81) = 6.93, *p *=* *.01. In the third step, introducing racial categorization scores further significantly increased explained variance by 9%, *F*(1, 80) = 8.69, *p *=* *.004. Racial categorization scores contributed significantly to the prediction of *D* scores, over and above gender and age, β = .35, *p *=* *.004, part correlation = .30, while age was no longer a significant predictor once racial categorization scores were factored into the model, β = .11, *p *=* *.37, part correlation = .09. The inclusion of the explicit racial bias scores (choice scores) in the fourth step did not significantly add to the explained variance, Δ*R* ^2^ = .02, *F*(1, 79) = 1.59, *p *=* *.21. After adjustment for the number of predictors, the final model explained 14.5% of variance in *D* scores, *F*(4, 79) = 4.53, *p *=* *.002, with the only significant predictor being racial categorization scores, β = .34, *p *=* *.006, part correlation = .29. Specifically, higher racial categorization scores were associated with higher levels of implicit bias (Figure 1a).

**Table 1:** Summary of Hierarchical Multiple Regression Analysis for Variables Predicting *D* Scores in Experiment 1 With Chinese Children

Variables	*B*	*SE* (*B*)	β	Part *r*	Δ*R* ^2^
Step 1					
Gender	−.05	.14	−.04	−.04	.001
Step 2					
Age	.02	.01	.28*	.28	.08*
Step 3					
Racial categorization scores	.11	.04	.35**	.30	.09**
Step 4					
Choice scores	.30	.24	.13	.13	.02

*p < .05. **p < .01.

#### Hierarchical Multiple Regression for Explicit Racial Bias

A hierarchical multiple regression was conducted on choice scores, with gender (Step 1), age (Step 2), racial categorization scores (Step 3), and *D* scores (Step 4) added sequentially to the model in four steps. The final model was nonsignificant, *F*(4, 79) = 1.88, *p *=* *.12, and none of the predictors significantly predicted choice outcome, all *p*s > .05. Racial categorization scores did not significantly predict choice outcome, β = .04, *p *=* *.77, part correlation = .03.

In summary, Experiment 1 examined implicit and explicit racial biases among Chinese Singaporean preschoolers who belonged to the racial majority in Singapore. We found evidence of significant pro‐Chinese implicit bias in the IRBT and explicit bias in the choice task. More importantly, we found support for our main hypothesis that the tendency of Chinese Singaporean preschoolers to reliably categorize people into racial groups is positively correlated with the strength of their implicit, but not explicit, racial bias.

## Experiment 2

We administered the same procedure as that in Experiment 1 to Indian preschoolers who attended Indian preschools in Singapore. Our aim was to examine whether the findings of Experiment 1 could be replicated with children who belong to a racial minority. Prior studies have reported that children from a racial minority (e.g., Black American) did not exhibit implicit racial bias against a racial majority (e.g., White; Dunham et al., 2013; Newheiser, Dunham, Merrill, Hoosain, & Olson, 2014). Additionally, racial minority children exhibited explicit racial bias against the racial majority group despite their lack of implicit racial bias against the racial majority (Newheiser & Olson, 2012). On the basis of these findings, we hypothesized that the Indian children in our study would manifest explicit but not implicit racial bias against Chinese. Alternatively, because the Indian children in our study attended racially homogeneous private schools, their daily exposure to predominantly own‐race individuals might lead them to show both implicit and explicit racial biases against Chinese, similar to the findings of implicit and explicit racial biases of Chinese children against Indians in Experiment 1.

### Method

#### Participants

The sample consisted of 71 preschool children aged 41–80 months old (38 male, 33 female; *M*_age_ = 60.63 months, *SD *= 9.75 months). Data collection took place over the period from February 2016 to October 2016. All participants were Indian, and 58.8% had at least one parent who is either a citizen or permanent resident of Singapore, whereas the remaining 41.2% had parents who were neither citizens nor permanent residents of Singapore. Parental consent was obtained prior to the study. Similar to Experiment 1, participants were recruited from Indian preschools located in the northern and eastern regions of Singapore, within housing estates mainly occupied by middle‐class families. Demographic characteristics of families residing in these regions are similar to those in Experiment 1. Consent forms were distributed to the preschools and children whose parents gave consent participated in a short testing session at their respective preschool.

#### Materials and Procedure

We used the same materials and procedure as in Experiment 1 except that on the congruent trials of the IRBT, participants were told to touch the cartoon frown when they saw a Chinese face and to touch the cartoon smile when they saw an Indian face, and the opposite was true for the incongruent trials.

### Results

#### Racial Categorization Task

Children's average score on the racial categorization task (*M *=* *6.10, *SD* = 1.68) was significantly higher than expected by chance (test value = 4), *t*(70) = 10.56, *p *<* *.001, Cohen's *d *=* *1.25. This result suggests that Indian preschoolers were able categorize faces based on race at an above‐chance level.

To assess for the possibility of a learning effect due to feedback on the task, a GLMM was conducted on children's categorization performance across the eight trials, with children's accuracy coded as a binary response (0 = incorrect, 1 = correct), trials as the fixed effect, and child ID entered as a random effect to account for repeated measures. Similar to Experiment 1, there was no evidence of learning across trials, β = −.06, *p *=* *.23.

#### Implicit Racial Bias

*D* scores were calculated in the same way as in Experiment 1. Participants with ≥ 10% of responses faster than 300 ms, an error rate > 80%, or an average response latency 3 *SD* above the mean response latency of the whole sample were excluded (*n* = 1). In addition, data from another four participants had to be excluded due to teacher or friend interference during the IAT test phase (*n* = 2), a child's refusal to follow the rule of using both hands to operate the tablet (*n* = 1), and a child running away during the IAT test phase (*n* = 1), leaving 66 participants for subsequent analyses.

A one‐sample *t* test revealed that the mean *D* score did not differ significantly from the no‐bias score of zero, *D *=* *−.003 (*SD *= 0.58), *t*(65) = −0.05, *p *=* *.96, Cohen's *d *=* *.01. Indian children were no faster at associating Indian faces with smile stimuli and Chinese faces with frown stimuli on the congruent trials than they were at associating Indian faces with frown stimuli and Chinese faces with smile stimuli on the incongruent trials. This finding suggests that Indian preschoolers as a group do not have a pro‐Indian implicit bias.

Additionally, *D* scores were computed separately for male and female face stimuli. A one‐way repeated measures ANOVA found no significant difference between *D* scores for male and female faces, *F*(1, 64) = 0.99, *p *=* *.32, partial η^2^ = .02.

#### Explicit Racial Bias

Choice scores were calculated as the proportion of trials in which each participant chose Indian over Chinese as their potential interaction partners. A one‐sample *t* test using the test value of 0.5 indicated that the children did not favor Indian over Chinese on a significantly greater proportion of trials (*M *=* *0.55, *SD* = 0.28) than chance, *t*(70) = 1.41, *p *=* *.16, Cohen's *d *=* *.17. This finding suggests that Indian preschoolers as a group did not exhibit a pro‐Indian explicit racial bias.

To further examine choice patterns on each scenario, binomial tests were conducted on choice responses in each scenario. Children were equally likely to choose Chinese (other race) or Indian (own race) in all scenarios, therefore exhibiting no explicit preference for own race, binomial *p*s > .05, except in Scenario 2 (in their choice of a dance teacher) where they were more likely to favor Indian (own race) over Chinese (other race), binomial *p *=* *.001.

#### Hierarchical Multiple Regression for Racial Categorization

Hierarchical multiple regression was conducted with racial categorization scores entered as the dependent variable. The data met the assumptions of independent, normally distributed errors, homogeneity of variance, and linearity, and contained no outliers or multicollinearity between predictors. In the first step of the regression model, gender was entered as a predictor. It accounted for a nonsignificant proportion of variance, Δ*R* ^2^ = .00, *F*(1, 66) = 0.001, *p *=* *.98. Entering age in the second step explained an additional 17.4% of the variance in racial categorization scores, and this increase in explained variance was significant, *F*(1, 65) = 13.67, *p *<* *.001. Age contributed significantly to the prediction of racial categorization scores, β = .42, *p *<* *.001, part correlation = .42.

#### Hierarchical Multiple Regression for Implicit Racial Bias

Hierarchical multiple regression was conducted on the *D* scores in order to identify the predictors of implicit racial bias. Checks were made to ensure that the data met all assumptions for the analysis. An examination of standard residuals confirmed that the data contained no outliers (std. residual min = −2.40, std. residual max = 2.45). Collinearity statistics of all predictor variables across all models were within acceptable limits (tolerance values > .10; VIFs < 10). The data also met the assumption of independent errors (Durbin–Watson value = 2.26). Furthermore, scatter plots of standardized residuals revealed that assumptions of normally distributed errors, homogeneity of variance, and linearity were satisfied.

Table 2 provides a summary of results from the hierarchical multiple regression analysis. Gender was entered as a predictor in the first step of the regression model. It accounted for a nonsignificant proportion of variance, Δ*R* ^2^ = .003, *F*(1, 61) = 0.17, *p *=* *.68. Entering age in the second step explained an additional 5.3% of the variance in *D* scores. This increase in explained variance was also nonsignificant, *F*(1, 60) = 3.38, *p *=* *.07. In the third step, introducing racial categorization scores further increased explained variance by 6.4%, *F*(1, 59) = 4.30, *p *=* *.04. Racial categorization scores contributed significantly to the prediction of *D* scores, over and above gender and age, β = .28 *p *=* *.04, part correlation = .25. The inclusion of the explicit racial bias scores (choice scores) in the fourth step did not significantly add to explained variance, Δ*R* ^2^ = .00, *F*(1, 58) = 0.01, *p *=* *.91. After adjustment for the number of predictors, the final model explained 5.9% of variance in *D* scores, *F*(4, 58) = 1.98, *p *=* *.11, with the only significant predictor being racial categorization scores, β = .28, *p *=* *.045, part correlation = .25. Higher racial categorization scores predicted higher levels of implicit bias (Figure 1b).

**Table 2:** Summary of Hierarchical Multiple Regression Analysis for Variables Predicting *D* Scores in Experiment 2 With Indian Children

Variables	*B*	*SE* (*B*)	β	Part *r*	Δ*R* ^2^
Step 1					
Gender	.06	.15	.05	.05	.003
Step 2					
Age	.01	.01	.23	.23	.05
Step 3					
Racial categorization scores	.10	.05	.28*	.25	.06*
Step 4					
Choice scores	.03	.30	.02	.01	.00

*p < .05.

#### Hierarchical Multiple Regression for Explicit Racial Bias

Hierarchical multiple regression was conducted on choice outcome, with gender (Step 1), age (Step 2), racial categorization scores (Step 3), and *D* scores (Step 4) added sequentially to the model in four steps. Gender significantly predicted choice outcome across all blocks, *p*s < .001. The final model was significant, *F*(4, 58) = 5.21, *p *=* *.001, with gender as the only significant predictor of choice outcome, β = .51, *p *<* *.001, part correlation = .51. Specifically, being female was associated with greater pro‐Indian bias in choice of interaction partners. Racial categorization scores did not significantly predict choice outcome, β = .06, *p *=* *.65, part correlation = .05.

In summary, Experiment 2 examined implicit and explicit racial biases among Indian preschoolers who belonged to a racial minority in Singapore. We found that Indian preschoolers as a group displayed neither significant pro‐Indian implicit bias nor pro‐Indian explicit bias in the choice task. More importantly, we found support for our main hypothesis that the tendency of Indian preschoolers to reliably categorize people into racial groups positively predicted their strength of implicit racial bias, but not that of explicit racial bias.

## General Discussion

The present study examined the relation between racial categorization performance and implicit racial bias among preschoolers who belong to either a racial majority (Chinese) or minority (Indian) in a multicultural society (Singapore). Several major findings were obtained. First, both Chinese and Indian preschoolers performed significantly better than chance in their racial categorization of Chinese and Indian faces. Second, there was significant positive implicit racial bias toward own race in Chinese preschoolers who belong to the racial majority in Singapore but not in Indian preschoolers who belong to a racial minority in Singapore. Third, the stronger the children's racial categorization performance, the greater their implicit racial bias. Fourth, Chinese but not Indian preschoolers exhibited strong explicit racial bias in favor of their own race. Finally, children's explicit racial bias was neither correlated with their implicit racial bias nor with their racial categorization performance. We will discuss these major findings in sequence.

### Differential Effects of Age on Racial Categorization and Bias

First, as expected, preschoolers in Singapore as a group were able to categorize faces by race, although there existed considerable variability in racial categorization performance. There was also a significant effect of age, such that older preschoolers were more reliable at assigning correct racial categories to face stimuli. These findings are consistent with prior reports that perceptual classification of faces by race becomes more robust with increasing age (Bigler & Liben, 1993; Dunham et al., 2013; Quintana, 1998; see also Lee et al., 2013; Pauker, Williams, & Steele, 2016 for reviews). On the other hand, we found no significant association between age and implicit or explicit racial bias in either the Chinese or Indian samples, after controlling for racial categorization performance. The absence of age‐related changes in racial biases among preschool children is consistent with the existing findings (e.g., Qian et al., 2016), suggesting that such biases take root early in childhood, do not naturally weaken with development, and may persist into adulthood (Baron & Banaji, 2006; Dunham et al., 2006). Taken together, the evidence highlights the importance of interventions to target biases when they first emerge in children as young as 3 years of age.

### Implicit Racial Bias: Majority Versus Minority Group

The second major finding that ethnic Chinese Singaporean preschoolers displayed an implicit racial bias favoring own race is consistent with findings with preschoolers and elementary school children in other countries (Baron & Banaji, 2006; Dunham et al., 2006; Qian et al., 2016). For example, Qian et al. (2016) found robust implicit racial bias for own race (Chinese) and against other race (Black) among Chinese preschoolers from mainland China that is, unlike Singapore, racially homogeneous. They also found that Black preschoolers in Cameroon manifested robust implicit racial bias for own race (Black) and against other race (Chinese). The present results contribute uniquely to the existing literature by providing the first indication that racial majority preschool children in a racially diverse society hold implicit racial bias despite the fact that the society is apparently racially harmonious and various social policies and practices have been specifically implemented to facilitate social harmony and interracial interaction. Taken together, the findings from this and previous studies suggest that children from the racial majority have a pro‐own‐race implicit bias that is already robust in early childhood and is perhaps little affected by social policies and societal practices in their environment.

However, we did not find a significant implicit bias favoring own race among ethnic Indian preschoolers who belong to a racial minority in Singapore. This is therefore one of the first studies to reveal that racial minority preschoolers do not exhibit implicit racial bias, although earlier studies have found similar results with older children and adults (Dunham et al., 2007; Newheiser & Olson, 2012; Nosek, Banaji, & Greenwald, 2002). The present data also converge with Dunham et al.'s (2013) finding that observed no significant implicit racial bias against White Americans in Black American children as measured by the “angry = out‐group” paradigm. A major explanation offered to account for the lack of implicit racial bias among racial minority individuals in previous studies has been the difference in socioeconomic status between the racial minority and the racial majority. Indeed, the racial minority groups targeted in prior studies tended to have relatively lower socioeconomic status than the racial majority groups (Dunham et al., 2007). However, this explanation does not apply to the present study because Indians in Singapore have comparable socioeconomic status to Chinese in Singapore (Singapore Department of Statistics, 2016).

The perceptual–social linkage hypothesis offers a possible alternative explanation for the difference in magnitude of implicit racial bias between the majority and minority children—namely, the opportunity for exposure to other‐race individuals. For children from the racial majority, opportunity for contact with other‐race individuals may be more limited than that for children from a racial minority. As a result, children from the majority group have greater asymmetry in experience with own‐race versus other‐race individuals, which may translate not only into greater perceptual processing differences between own‐ and other‐race faces, but also more pronounced racial bias. In contrast, children from the racial minority, by virtue of their being a racial minority, have greater opportunity to be exposed to individuals from the racial majority. Consequently, they show no implicit racial bias favoring the minority own race and against the majority other race.

### Racial Categorization and Implicit Racial Bias

Our third major finding supports and extends the perceptual–social linkage hypothesis as a framework for our study. According to the hypothesis, children's tendency to categorize faces by race develops as a result of asymmetrical exposure to own‐ and other‐race faces (Quinn et al., 2016), and this tendency in turn leads to implicit racial bias for the highly familiar own race and against the unfamiliar other race. Consistent with the hypothesis, we found that among Chinese preschoolers, those with greater categorization accuracy for Chinese and Indian faces had stronger implicit racial bias. Similarly, although the Indian preschoolers as a group did not exhibit an implicit bias for own race and against the racial majority (Chinese), they still showed considerable variability in implicit racial bias scores. Some showed strong pro‐Indian implicit bias while others did not. Such variability in implicit racial bias was significantly correlated with performance in categorizing own‐ and other‐race faces. That is, the better the children could categorize Chinese and Indian faces by race, the stronger their implicit racial bias for own race and against other race. The findings thus provide evidence for a connection between categorization of face race stimuli (using labels) and implicit racial bias.

Our findings are consistent with those of Dunham et al. (2013) showing that the *other race = angry* bias in White American and Taiwanese children was related to their performance in categorizing faces by race: Controlling for age, children who categorized unambiguous White or Asian faces correctly were more likely to categorize racially ambiguous angry faces as belonging to the racial out‐group, whereas children who were unable to successfully categorize by race were unlikely to exhibit the *other race = angry* bias. As noted in the Introduction, in Dunham et al. (2013), both the categorization and implicit bias measures came from the same task (i.e., categorizing faces by race) and the faces were synthetic, whereas in the present study, the categorization and implicit bias measures came from two different tasks and the faces were natural. Despite these methodological differences, both sets of data converge to suggest that for children from both majority and minority groups, the readiness to use perceptual information of faces to classify them into racial groups may be an important basis from which children's implicit racial bias emerges and develops.

### Explicit Bias: A Role for Social Status

Our fourth major finding is that Chinese preschoolers in Singapore displayed strong explicit bias favoring own race. This finding is consistent with existing studies on preschool children's explicit racial bias in which racial majority children show explicit racial bias in choosing own‐race interaction partners (e.g., Fishbein, 2002). The finding, however, differs from data indicating that racial majority Black children in South Africa show an explicit preference for racial minority White children (Shutts et al., 2011). These seemingly contradictory outcomes point to the important role that social status plays in shaping children's explicit racial preferences: When the racial majority has a higher social status than the minority, racial majority children are biased to choose the own race with higher social status, whereas when the racial majority has lower social status, racial majority children are biased to choose the other race with higher social status. In accord with this suggestion, in the present study, when the racial majority had social status similar to the racial minority, racial minority Indian children were not biased overall.

### Implicit Versus Explicit Racial Bias

We also found that the children's explicit racial bias scores were uncorrelated with both their implicit racial bias scores and racial categorization scores. The dissociation between implicit and explicit racial biases in preschool children is corroborated by findings of such a dissociation among preschoolers from Africa and China (Qian et al., 2016), and is also in line with the conclusion of other studies with older children and adults (Augoustinos & Rosewarne, 2001; Dunham et al., 2006; Greenwald et al., 1998, 2003; Rutland, Cameron, Milne, & McGeorge, 2005). However, as pointed out by Qian et al. (2016), whereas older children and adults tend to show strong implicit racial bias but no explicit racial bias, preschoolers show both implicit and explicit biases. Thus, the lack of correlation between implicit and explicit racial biases is a robust finding in the literature with studies involving children from countries such as the United States, China, Japan, and Cameroon (Dunham et al., 2006; Qian et al., 2016). Furthermore, regardless of whether the child belongs to a racial majority or minority group, explicit and implicit racial biases are unrelated. It is possible that these two biases may operate on different sets of mechanisms.

It should be noted that methods of measuring explicit racial bias differ depending on the age of the participant. Studies with older children and adults typically employ scale ratings of race‐related beliefs (e.g., Greenwald et al., 2003). In contrast, studies with younger children adopt age appropriate measures such as forced‐choice preference, judgments of liking (Baron & Banaji, 2006; Dunham et al., 2007; Shutts et al., 2011), and negative versus positive trait attributions (Augoustinos & Rosewarne, 2001; Rutland et al., 2005). The absence of explicit racial bias in older children and adults has been attributed to social desirability (Greenwald et al., 1998; Rutland et al., 2005). Although some investigations suggest that preschool‐aged children respond in socially desirable ways in certain situations (Fu & Lee, 2007; Rutland et al., 2005), other research has found that social desirability stabilizes only at 11 years of age (Klein, Gould, & Corey, 1969), and there is little evidence that children younger than 7 years of age adhere to social desirability norms in responding to intergroup scenarios (Heiphetz, Spelke, & Banaji, 2013; Rutland et al., 2005). Social desirability may therefore be one plausible explanation to account for the lack of strong explicit biases in older children and adults. In contrast, unlike their older counterparts, young children may not suppress or mask their overt in‐group preferences in order to present themselves in line with social norms of fairness and racial inclusivity. Indeed, racial majority preschool children in many countries (including Singapore) display strong explicit racial biases.

Another possible explanation for the lack of association between implicit and explicit racial biases in preschool children is that different experiences may differentially affect the formation of the two forms of racial bias (Qian et al., 2016). Although implicit racial bias may be influenced more by children's asymmetrical perceptual processing experiences with own‐ and other‐race faces (Lee et al., 2017), children's explicit racial bias may be more influenced by children's interactions with various socialization agents such as parents, educators, and peers (see Allport, 1954). By this explanation, children's explicit racial bias should be most closely related to that of individuals with whom they have extensive social interaction, such as parents (see Degner & Dalege, 2013). Consistent with this idea are the results of a recent survey regarding the racial attitudes of adults in Singapore (Mathews, 2016). The survey found that Singaporean Chinese prefer a Singaporean Chinese over a Singaporean Indian for the position of Singapore's Prime Minister (98% vs. 60%). Singaporean Indians were less pro‐own race; they showed equal preference for a Singaporean Indian or Singaporean Chinese Prime Minister (89% vs. 88%). These adult preferences are mirrored in the explicit choices of the Chinese and Indian children we tested. Chinese children displayed explicit bias in favor of their own racial group, whereas Indian children did not favor one group over another.

### Limitations and Future Directions

There are some limitations of the present work that point to important future directions. First, the current study is correlational in nature, which precludes drawing causal inferences from the results. The perceptual–social linkage hypothesis proposes that increased racial categorization performance facilitates heightened implicit racial bias. Experimental studies that support this suggestion reported that training children to individuate (rather than categorize) other‐race faces reduces implicit racial bias (Qian et al. 2017; Xiao et al., 2015). However, it is also possible that children who have high levels of implicit racial bias become more attuned to the race of others and hence develop greater accuracy at classifying race. Future studies can provide a clearer picture of the possibly bidirectional relation between racial categorization and implicit racial bias by adopting a longitudinal design that examines the relations between the two variables from infancy to preschool.

Second, the racial categorization task in the present study demands some knowledge and familiarity with verbal race labels, such that performance on the task could be related to linguistic factors that occur outside of purely perceptual processes. For instance, although some scholars have contended that children may not understand the correspondence between racial labels and categories (Hirschfeld, 1996), more recent work has suggested that children who are perceptually able to categorize faces by race also understand how labels correspond to the categorical distinction between races (Waxman, 2010). Future studies can provide a stronger test of the perceptual–social linkage hypothesis by using a nonverbal racial categorization task to minimize the effect of race labels on task performance.

Third, although the present study has revealed a significant relation between children's racial categorization and their implicit racial bias, additional work is needed to further elucidate the relation. For example, the present study targeted two of the four main racial groups in Singapore. Future studies could explore implicit and explicit racial biases of Chinese and Indian preschoolers against the other two racial groups (i.e., Malay and Eurasian) to ascertain whether Chinese preschoolers have general biases against all other racial groups and whether Indian preschoolers, although not having an implicit bias against the Chinese majority, still hold negative biases against the other racial minorities. Work is also needed to determine whether racial categorization performance for one out‐group race translates to comparable performance for all out‐group races or whether such performance is race specific. Finally, it is unclear whether Chinese and Indian preschoolers will show high variability in categorizing Malay and Eurasian faces and whether any such variability is related to implicit racial biases against these racial groups. Addressing these questions will help to elucidate the generalizability of the present findings about racial categorization, racial biases, and the relation between the two in preschool children.

Fourth, the implicit and explicit racial bias measures used in this study may reflect a positive in‐group bias, negative out‐group bias, or both positive in‐group and negative out‐group biases. In‐group preference on its own does not automatically equate to out‐group dislike or translate into overt acts of prejudice and discrimination (e.g., Nesdale, Maass, Durkin, & Griffiths, 2005). Given that the IRBT collapses across the two types of biases, and the explicit choice task involves choosing a partner for positive interactions, neither measure reveals whether the nature of children's racial biases is one that is solely in favor of the in‐group, or if it involves negativity toward the out‐group as well. Future studies can use IRBTs that measure children's implicit pro‐own race bias and implicit anti‐other race bias separately. Such measures will allow for deeper delineation of the link between their implicit racial biases and their skills in perceptual categorization of faces by race. Regarding explicit racial bias, the current measure needs to be modified so as to assess both children's explicit pro‐own race bias and anti‐other race bias. For example, we can assess the extent of children's negative racial biases against the out‐group by incorporating a wider scope of tasks such as examining preferences for delegating unpleasant jobs to same‐ versus other‐race members. Studies using these types of explicit racial bias measures are particularly important for addressing the discrepancies between the present finding and existing findings concerning the relation between social categorization and explicit bias.

Finally, an important field of inquiry that extends from the present study concerns how the quality and quantity of social experience may play a pivotal role in shaping young children's understanding of racial categories (Waxman, 2012). Singapore offers a unique testing ground for investigating such influences due to its diverse social environment, social policies and initiatives that staunchly inculcate the importance of racial harmony, and high levels of integration among racial groups in the macroenvironment. In addition, natural variation exists in the opportunities for frequent, prolonged, and personal interactions with other races in the child's microenvironment, depending on arrangements for out‐of‐home care (e.g., racial heterogeneity of preschools) and home care (e.g., family employment of other‐race caretakers). Therefore, future studies could examine the correlates of racial categorization performance by capturing such variabilities in social experience. Moreover, cross‐cultural studies that allow for comparisons with more racially homogenous societies, or with highly segregated multiracial communities that are characterized by interracial tensions, will offer valuable insight into how different facets of social experience interact to influence the development of racial categorization, the valence attached to racial categories, and related implicit and explicit racial biases.

### Translating Findings Into Practice

The major findings of the present study have not only important theoretical implications in terms of understanding how and why implicit and explicit racial biases emerge and develop in children but also carry practical implications. For example, our findings may aid in the design of multicultural and multiracial curricula in schools. In communities that consist of diverse racial and ethnic groups, promoting intergroup harmony can be challenging but is essential. Although school programs often employ explicit teaching about races with the aim of educating children about the diversity of their community, empirical support for such programs is limited (Bigler, 1999). In particular, existing pedagogical approaches tend to involve teaching children how to differentiate races and encourage the use of linguistic labels that sort people into racial categories (Patterson & Bigler, 2006; Pauker et al., 2016). Such teaching increases the salience of racial categories (e.g., Waxman, 2010), which according to the present findings may be counterproductive given that the readiness to categorize by race is associated with heightened implicit racial biases in children.

The main finding of a linkage between racial categorization and implicit racial bias points to alternative approaches that can break down this connection. As noted earlier, one approach that has been successful in reducing implicit racial bias with preschool children is training to individuate other‐race faces (Qian et al., 2017; Xiao et al., 2015). This method capitalizes on the finding that other‐race recognition is negatively correlated with the tendency to categorize such faces (Ge et al., 2009). A likely reason then for the bias reduction is that individuation training, while increasing one's tendency to recognize and differentiate other‐race faces, also reduces the automatic tendency to categorize other‐race faces as the racial out‐group.

### Conclusion

To conclude, the present studies have revealed that racial majority preschool children from a racially diverse society show implicit and explicit biases for own race and against a racial minority group, whereas children belonging to a racial minority group of similar socioeconomic status display neither explicit nor implicit bias against the racial majority. We also found that children's implicit, but not explicit, racial bias is significantly correlated with their performance in categorizing faces by race, which supports the perceptual–social linkage hypothesis. Our main finding of the association between racial categorization of faces and implicit racial bias suggests that we may need to rethink approaches to multicultural and multiracial education. Instead of the current emphasis on the uniqueness of different racial and ethnic groups and labeling them by categories, our findings support recommendations for a perceptual training approach that reduces children's automatic tendency to categorize faces by race.
